# Supplementary material for: Asynchronous release sites align with NMDA receptors in mouse hippocampal synapses
Source: Nat Commun. 2021 Jan 29;12:677. doi: 10.1038/s41467-021-21004-x (PMC7846561; doi:10.1038/s41467-021-21004-x)
Supplement: Supplementary file 1 — Supplementary Information [file 41467_2021_21004_MOESM1_ESM.pdf]

Supplemental figure 1, Li et al.

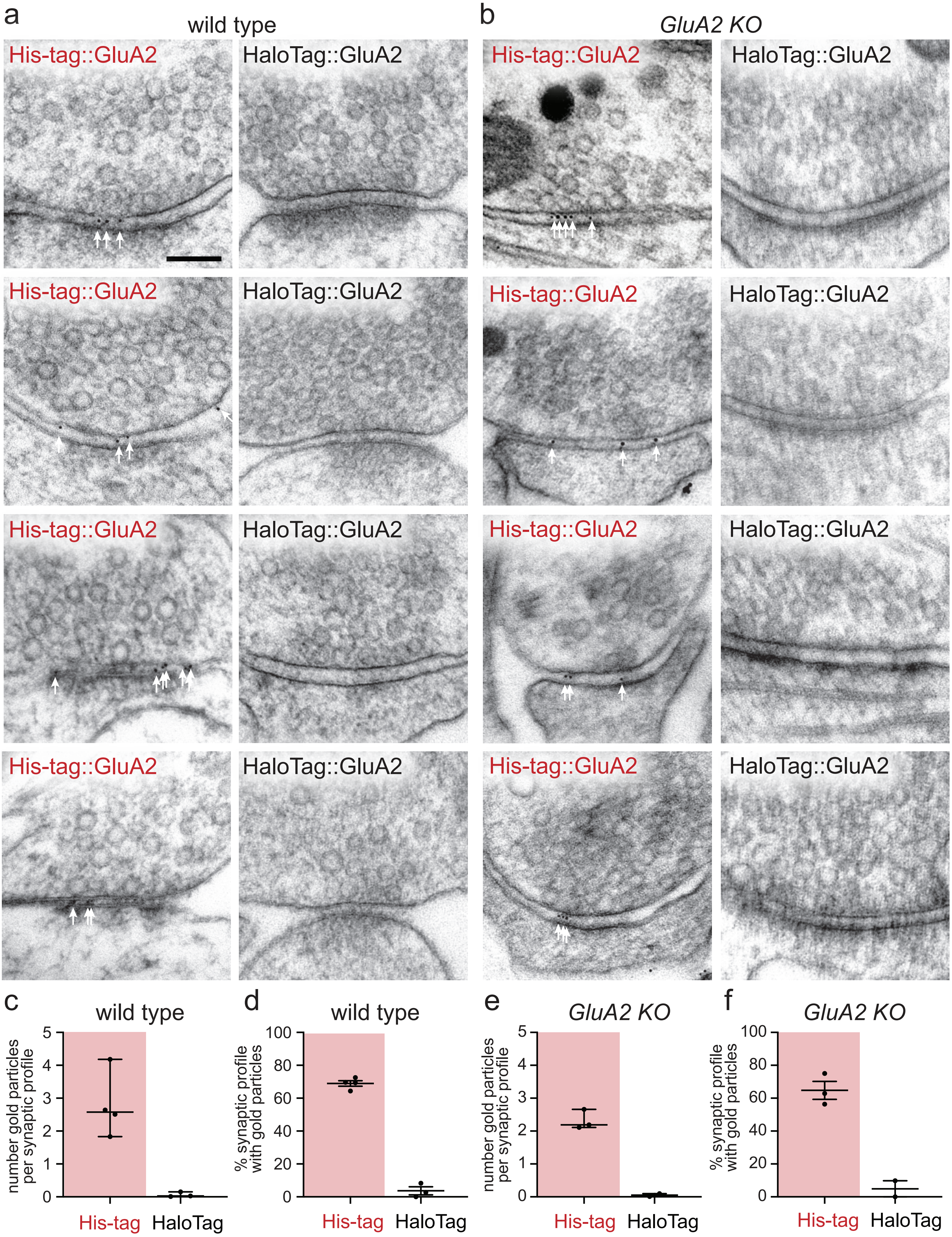

Supplementary Fig. 1. The receptor labelling approach and its validation

a-b, Example transmission electron micrographs of synapses after SMASH labelling and high-pressure freezing, showing gold particles in the synaptic cleft of wild-type (**a**) and *GluA2* knock-out (**b**, *Gria2*$^{-/-}$) neurons expressing His-tag::GluA2. Scale bar: 100 nm. White arrows indicate gold particles. **c**, Number of gold particles per synaptic cleft in the wild-type neurons. Each dot: an average number from a single experiment, analyzing ~100 micrographs. Error bars: median and 95 % confidence interval, $p=0.01$, Welch's T-test. **d**, Percentage of synaptic profiles containing gold particles in synaptic cleft in the wild-type neurons. Each dot: a percentage from a single experiment, analyzing ~100 micrographs. Error bars: mean and SEM, $p<0.001$, Welch's T-test. **e**, Same as in **c**, except showing the number from the *GluA2* knock-out (KO) neurons. Error bars: median and 95% confidence interval, $p<0.01$, Welch's T-test. **f**, Same as in **d**, except showing the number from the *GluA2* knock-out (KO) neurons. Error bars: mean and SEM, $p<0.01$, Welch's T-test.

Supplemental figure 2, Li et al.

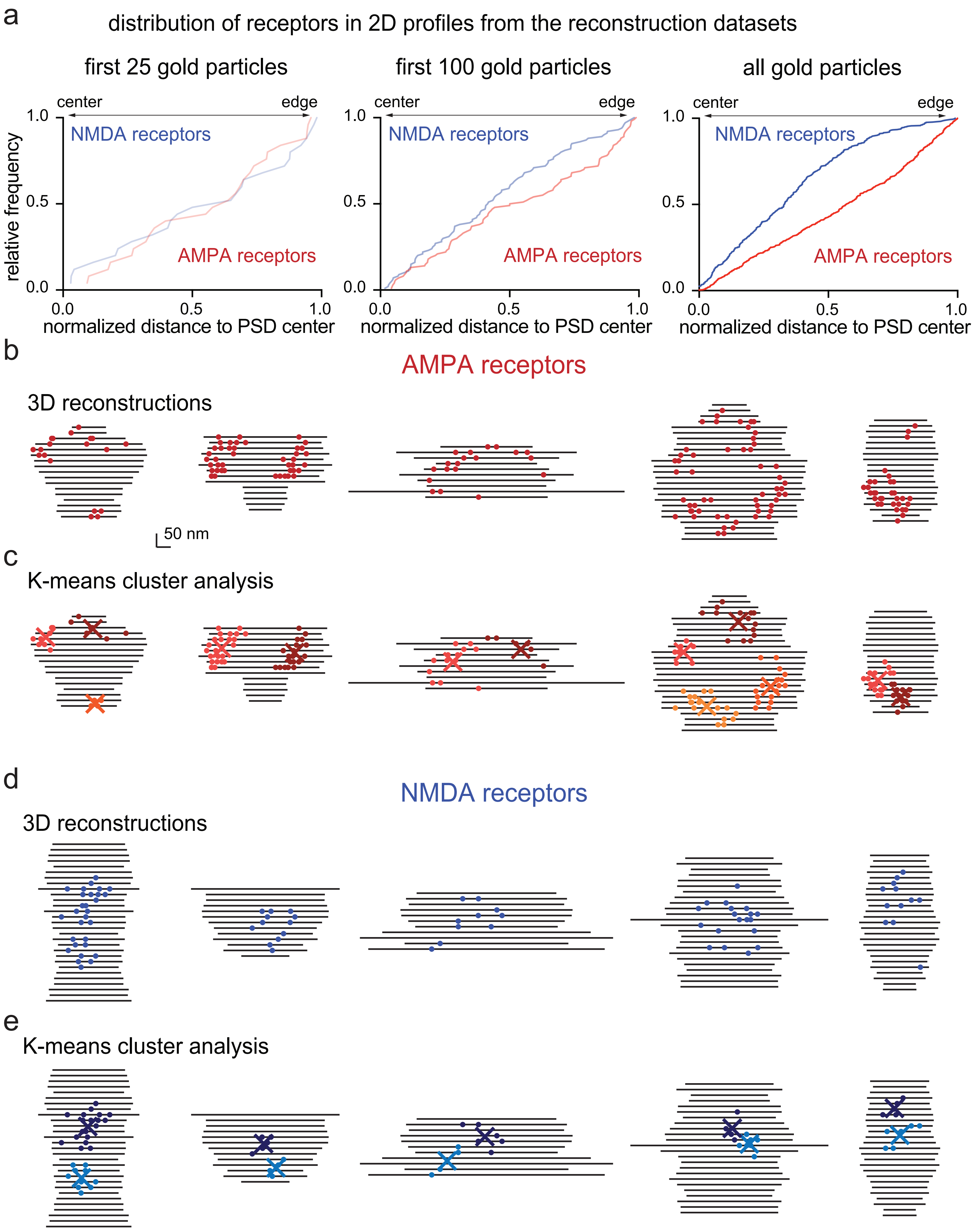

Supplementary Fig. 2. Additional 3-D representations of AMPA receptors and NMDA receptors.

a, Cumulative relative frequency distribution of lateral distances from gold particles to the center of the postsynaptic density (PSD) from synaptic profiles. The 3-D reconstruction dataset in **Fig. 2b** is used. Distances are normalized to the length of the PSD: a gold particle at 0 would be exactly at the center and at 1 exactly at the edge. AMPA receptors are biased towards the edge, while NMDA receptors are biased towards the center when all gold particles are analyzed (AMPA receptors: mean = 0.55, n = 25; mean = 0.54, n = 100; mean = 0.55, n = 808 particles; NMDA receptors: mean = 0.55, n =25; mean = 0.44, n = 100; mean = 0.35, n = 551 particles). **b**, Examples of synapses of neurons expressing His-tag::GluA2 from spin-mill serial block face scanning electron microscopy; each line indicates the extent of the cleft in a single 20 nm-thick 2-D profile, each circle indicates the location of a gold particle. **c**, Same as **b,** except showing the centers of clusters determined by k-means clustering. **d**, Examples of synapses of neurons expressing His-tag::NR1 from 3-D serial imaging; each line indicates the extent of the cleft in a single 20 nm-thick 2-D profile, each circle indicates the location of a gold particle. **e**, Same as **d,** except showing the centers of clusters determined by k-means clustering.

Supplemental figure 3. Li et al.

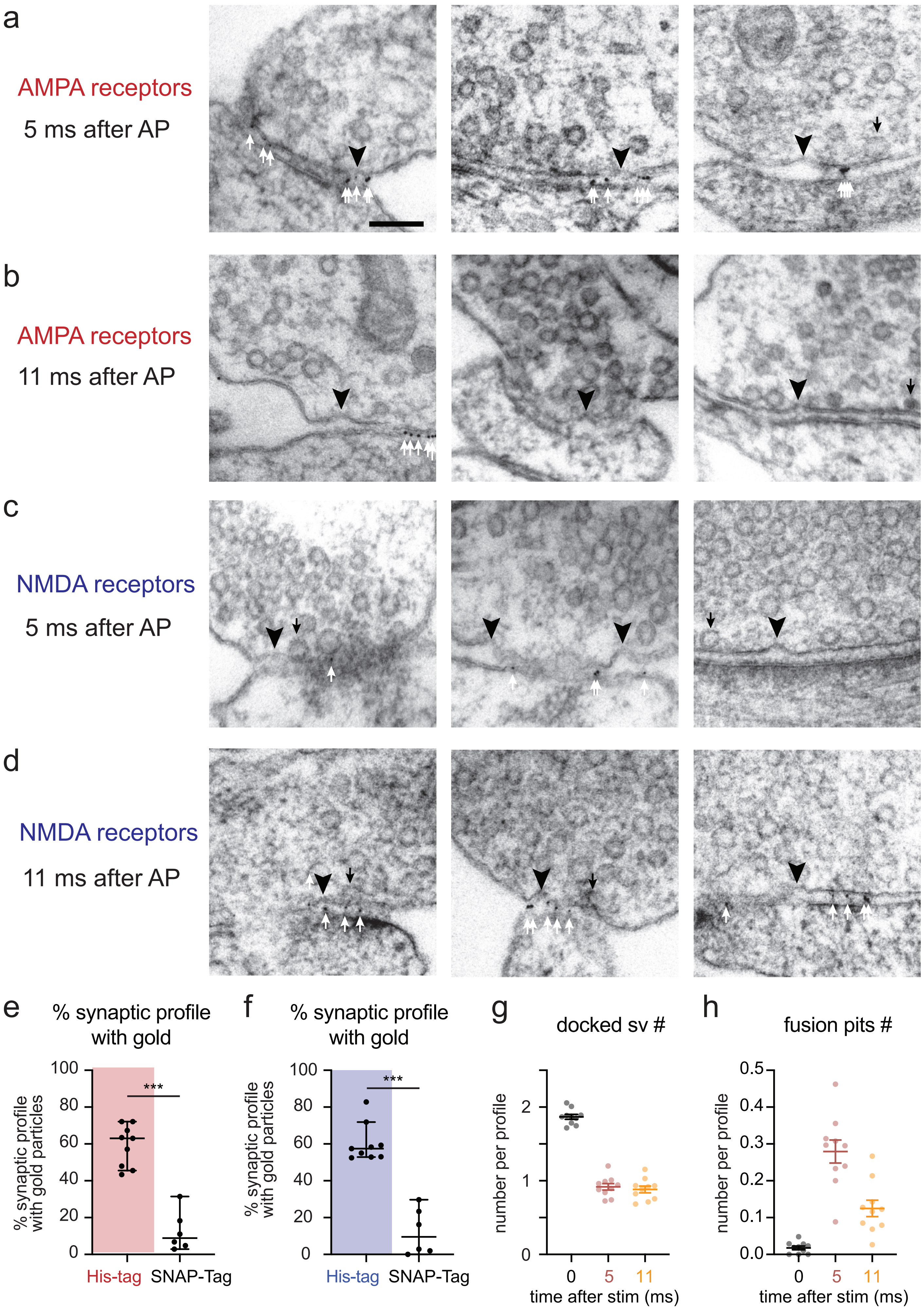

Supplementary Fig. 3. Synchronous and asynchronous release are aligned to AMPA receptors and NMDA receptors, respectively.

a-d, Additional example transmission electron micrographs of synapses after SMASH labelling and high-pressure freezing at 5 ms (**a, c**) and 11 ms (**b, d**) post stimulus (1-ms pulse), showing pits (black arrowheads), docked vesicles (black arrows) and gold particles (white arrows) at synapses of wild-type neurons expressing His-tag::GluA2 (**a-b**) and His-tag::NR1 (**c-d**). **e-f**, Percentage of synaptic profiles containing gold particles in synaptic cleft in the wild-type neurons expressing His-tag::GluA2 or SNAP-tag::GluA2 (**e**) and His-tag::NR1 or SNAP-tag::NR1 (**f**). Each dot: a percentage from a single experiment, analyzing ~100 micrographs. Error bars: mean and SEM, $p<0.001$, Welch's T-test. **g-h**, Number of docked vesicles (**g**) and pits (**h**) in the active zone per synaptic profile from the neurons either unstimulated (black) or stimulated once (a 1-ms pulse) 5 ms (red) or 11 ms (orange) before freezing. Each dot: a mean from a single experiment, analyzing ~100 micrographs. Error bars: mean and SEM, $p<0.001$ in all cases except for the numbers of docked vesicles between 5 and 11 ms ($p>0.6$), 2-way ANOVA with post hoc Turkey's multiple comparisons test.

Supplemental figure 4. Li et al.

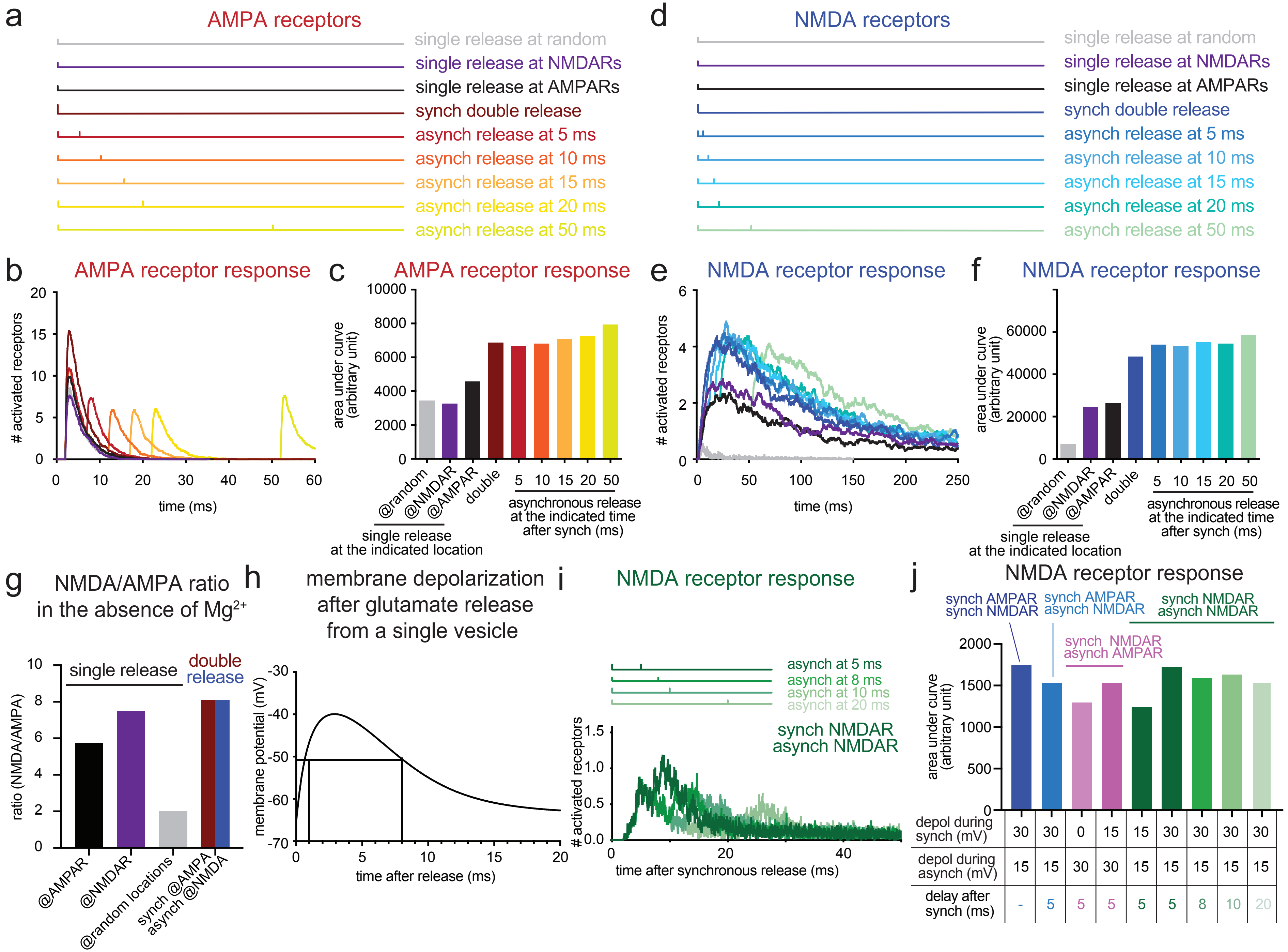

Supplementary Fig. 4. Computer simulations predict better activation of NMDA receptors with asynchronous release.

a-b, Time course of simulated AMPA receptor activation in the absence of Mg^{2+} (**b**), resulting from release events at the timing and locations as indicated in (**a**) and (**b**), respectively. The vertical lines in (**a**) indicate when synchronous and asynchronous release was applied. The number averaged from 48 simulations are plotted in (**b**). The synchronous release was applied at time 0. Double release means two release events were applied simultaneously. Synch = synchronous. Asynch = asynchronous. **c**, the area under curve calculated from each dataset in (**b**) and plotted as a bar graph. **d-e**, same as in **a-b**, but for NMDA receptors. **f**, same as in **c** but for NMDA receptors. **g**, NMDA/AMPA ratio calculated from the data in (**c**,**f**) and plotted as a bar graph. **h**, Time course of simulated membrane depolarization due to the activation of AMPA receptors. The depolarization peaks between 3 to 5 ms after the glutamate release, declines to 2/3 of the maximum after 8 ms, and is almost back to the baseline by 10 ms. **i**, Time course of simulated NMDA receptor activation in the presence of Mg^{2+}, resulting from both synchronous and asynchronous release occurring near the NMDA receptors. The membrane depolarization due to synchronous release was simulated at 30 mV. **j**, The area under curve calculated from each dataset in (**i**) and plotted as a bar graph. The locations of synchronous and asynchronous release are as indicated. The degree of depolarization (depol) and the delay between synchronous and asynchronous release used in simulated are listed at the bottom.

Supplementary Table 1.

**Fig. 1d. Cumulative relative frequency distribution of lateral distances from gold particles to the center of the postsynaptic density (PSD) from 2-D profiles.**		
	His-tag::GluA2	His-tag::NR1
N (samples)	4	4
Number of values	891	746

Minimum	0	0
25% Percentile	0.3717	0.2342
Median	0.6669	0.4847
75% Percentile	0.8618	0.7677
Maximum	0.9989	0.996
Range	0.9989	0.996
95% CI of median		
Actual confidence level	95.56%	95.60%
Lower confidence limit	0.6428	0.448
Upper confidence limit	0.6978	0.5244
Mean	0.6063	0.4977
Std. Deviation	0.2878	0.2946
Std. Error of Mean	0.009642	0.01079
Lower 95% CI of mean	0.5874	0.4766
Upper 95% CI of mean	0.6252	0.5189
Coefficient of variation	47.47%	59.18%
Skewness	-0.5142	0.03349
Kurtosis	-0.932	-1.27

**one sample Wilcoxon test (against a theoretical median)**		
Theoretical median	0.5	0.5
Actual median	0.6669	0.4847
Number of values	891	746
Wilcoxon Signed Rank Test		
Sum of signed ranks (W)	157596	-1925
Sum of positive ranks	277491	138353
Sum of negative ranks	-119895	-140278
P value (two tailed)	<0.0001	0.8702
Exact or estimate?	Approximate	Approximate
P value summary	****	ns
Significant (alpha=0.05)?	Yes	No
How big is the discrepancy?		
Discrepancy	0.1669	-0.01528
95% confidence interval	0.1428 to 0.1978	-0.05197 to 0.02440
Actual confidence level	95.56	95.6
**Kolmogorov-Smirnov test (against each other)**		
P value	<0.0001	
Exact or approximate P value?	Approximate	
P value summary	****	
Significantly different (P < 0.05)?	Yes	

Kolmogorov-Smirnov D	0.184	

Supplementary Table 2.

**Fig. 1f. Cumulative relative frequency distribution of lateral distances from gold particles to the center of the postsynaptic density (PSD) from 3-D profiles.**		
	His-tag::GluA2	His-tag::NR1
N (samples)	2	2
Number of values	840	550
Minimum	0	0
25% Percentile	0.2701	0.1492
Median	0.559	0.3306
75% Percentile	0.8075	0.5086
Maximum	0.9997	0.9908
Range	0.9997	0.9908
95% CI of median		
Actual confidence level	95.08%	95.50%
Lower confidence limit	0.5354	0.2958
Upper confidence limit	0.5988	0.3551
Mean	0.5358	0.3487

Std. Deviation	0.3009	0.2402
Std. Error of Mean	0.01038	0.01024
Lower 95% CI of mean	0.5154	0.3286
Upper 95% CI of mean	0.5562	0.3688
Coefficient of variation	56.17%	68.88%
Skewness	-0.1781	0.5744
Kurtosis	-1.263	-0.3541
**one sample Wilcoxon test (against a theoretical median)**		
Theoretical median	0.5	0.5
Actual median	0.559	0.3306
Number of values	840	550
Wilcoxon Signed Rank Test		
Sum of signed ranks (W)	47810	-93751
Sum of positive ranks	200515	28887
Sum of negative ranks	-152705	-122638
P value (two tailed)	0.0007	<0.0001
Exact or estimate?	Approximate	Approximate
P value summary	***	****
Significant (alpha=0.05)?	Yes	Yes

How big is the discrepancy?		
Discrepancy	0.05896	-0.1694
**Kolmogorov-Smirnov test**		
P value	<0.0001	
Exact or approximate P value?	Approximate	
P value summary	****	
Significantly different (P < 0.05)?	Yes	
Kolmogorov-Smirnov D	0.3163	

Supplementary Table 3.

**Fig. 1i. Median areas of postsynaptic density**		
	His-tag::GluA2	His-tag::NR1
N (samples)	2	2
Number of values	45	40
Minimum	24800	19380
25% Percentile	48220	41360

Median	68600	64810
75% Percentile	103850	113125
Maximum	318360	421740
Range	293560	402360
95% CI of median		
Actual confidence level	96.43%	96.15%
Lower confidence limit	64980	52640
Upper confidence limit	84080	87480
Mean	91228	92600
Std. Deviation	66974	86552
Std. Error of Mean	9984	13685
Lower 95% CI of mean	71107	64919
Upper 95% CI of mean	111349	120280
Coefficient of variation	73.41%	93.47%
Skewness	1.75	2.699
Kurtosis	3.005	8.168
**Unpaired t test with Welch's correction**		
P value	0.9357	

P value summary	ns	
Significantly different (P < 0.05)?	No	
One- or two-tailed P value?	Two-tailed	
Welch-corrected t, df	t=0.08096, df=73.19	
How big is the difference?		
Mean of column A	91228	
Mean of column B	92600	
Difference between means (B - A) ± SEM	1372 ± 16940	
95% confidence interval	-32388 to 35131	
R squared (eta squared)	0.00008956	
F test to compare variances		
F, DFn, Dfd	1.670, 39, 44	
P value	0.1	
P value summary	ns	
Significantly different (P < 0.05)?	No	
Data analyzed		
Sample size, column A	45	
Sample size, column B	40	
**Mann Whitney test**		
P value	0.6886	

Exact or approximate P value?	Exact	
P value summary	ns	
Significantly different (P < 0.05)?	No	
One- or two-tailed P value?	Two-tailed	
Sum of ranks in column A,B	1981 , 1674	
Mann-Whitney U	854	
Difference between medians		
Median of column A	68600, n=45	
Median of column B	64810, n=40	
Difference: Actual	-3790	
Difference: Hodges-Lehmann	-4770	

Supplementary Table 4.

**Fig. 1j. Number of clusters of receptors**		
	His-tag::GluA2	His-tag::NR1
N (samples)	2	2
Number of values	40	39
Minimum	0	0
25% Percentile	1	0

Median	2	1
75% Percentile	3	2
Maximum	5	5
Range	5	5
95% CI of median		
Actual confidence level	96.15%	97.63%
Lower confidence limit	1	0
Upper confidence limit	2	2
Mean	1.9	1.333
Std. Deviation	1.411	1.284
Std. Error of Mean	0.223	0.2056
Lower 95% CI of mean	1.449	0.917
Upper 95% CI of mean	2.351	1.75
Coefficient of variation	74.24%	96.31%
Skewness	0.1858	0.8237
Kurtosis	-0.8199	0.5656
**Unpaired t test with Welch's correction**		
P value	0.0656	

P value summary	ns	
Significantly different (P < 0.05)?	No	
One- or two-tailed P value?	Two-tailed	
Welch-corrected t, df	t=1.87, df=76.6	
How big is the difference?		
Mean of column A	1.9	
Mean of column B	1.33	
Difference between means (B - A) ± SEM	-0.567 ± 0.303	
95% confidence interval	-1.17 to 0.0374	
R squared (eta squared)	0.0435	
F test to compare variances		
F, DFn, Dfd	1.21, 39, 38	
P value	0.5642	
P value summary	ns	
Significantly different (P < 0.05)?	No	
Data analyzed		
Sample size, column A	40	
Sample size, column B	39	
**Mann Whitney test**		
P value	0.0572	

Exact or approximate P value?	Exact	
P value summary	ns	
Significantly different (P < 0.05)?	No	
One- or two-tailed P value?	Two-tailed	
Sum of ranks in column A,B	1787 , 1373	
Mann-Whitney U	593	
Difference between medians		
Median of column A	2.000, n=40	
Median of column B	1.000, n=39	
Difference: Actual	-1	
Difference: Hodges-Lehmann	-1	

Supplementary Table 5.

**Fig. 1h. Sum of squared differences**		
	actual data	randamized
N (samples)	2	
**simple linear regression for AMPA receptor data**		
Best-fit values		
Slope	0.1111	0.4758

Y-intercept	7199	8648
X-intercept	-64780	-18174
1/slope	8.998	2.102
Std. Error		
Slope	0.03124	0.06146
Y-intercept	3546	7049
95% Confidence Intervals		
Slope	0.04704 to 0.1752	0.3503 to 0.6014
Y-intercept	-76.37 to 14474	-5747 to 23043
X-intercept	-284850 to 470.8	-62854 to 10002
Goodness of Fit		
R squared	0.3192	0.6664
Sy.x	11518	23726
Is slope significantly non-zero?		
F	12.66	59.94
DFn, DFd	1, 27	1, 30
P value	0.0014	<0.0001
Deviation from zero?	Significant	Significant
Equation	Y = 0.1111*X + 7199	Y = 0.4758*X + 8648

Data		
Number of X values	40	42
Maximum number of Y replicates	1	1
Total number of values	29	32
Number of missing values	11	10
Are the slopes equal?	F = 26.33. DFn = 1, DFd = 57	
	P<0.0001	
**simple linear regression for NMDA receptor data**		
Best-fit values		
Slope	0.7289	1.224
Y-intercept	-28509	-21430
X-intercept	39112	17511
1/slope	1.372	0.8171
Std. Error		
Slope	0.1199	0.1943
Y-intercept	18046	28172
95% Confidence Intervals		
Slope	0.4802 to 0.9776	0.8228 to 1.625
Y-intercept	-65934 to 8916	-79573 to 36714
X-intercept	-16498 to 75908	-40257 to 54282

Goodness of Fit		
R squared	0.6267	0.6231
Sy.x	60011	99280
Is slope significantly non-zero?		
F	36.94	39.67
DFn, DFd	1, 22	1, 24
P value	<0.0001	<0.0001
Deviation from zero?	Significant	Significant
Equation	Y = 0.7289*X - 28509	Y = 1.224*X - 21430
Data		
Number of X values	39	39
Maximum number of Y replicates	1	1
Total number of values	24	26
Number of missing values	15	13
Are the slopes equal?	F = 4.560. DFn = 1, DFd = 46	
	P=0.0381	

Supplementary Table 6.

**Fig. 1k. Number of gold particles per cluster**		
	His-tag::GluA2	His-tag::NR1
N (samples)	2	2
Number of values	76	62
Minimum	4	4
25% Percentile	6	5
Median	8	6
75% Percentile	12	8.25
Maximum	23	18
Range	19	14
95% CI of median		
Actual confidence level	97.1%	97.0%
Lower confidence limit	7	5
Upper confidence limit	10	7
Mean	9.68	7.35
Std. Deviation	5.09	3.65
Std. Error of Mean	0.584	0.464
Lower 95% CI of mean	8.52	6.43
Upper 95% CI of mean	10.8	8.28

Coefficient of variation	52.6%	49.7%
Skewness	1.07	1.65
Kurtosis	0.333	2.29
Mann Whitney test		
P value	0.0024	
Exact or approximate P value?	Exact	
P value summary	**	
Significantly different (P < 0.05)?	Yes	
One- or two-tailed P value?	Two-tailed	
Sum of ranks in column A,B	5982 , 3610	
Mann-Whitney U	1657	
Difference between medians		
Median of column A	8.00, n=76	
Median of column B	6.00, n=62	
Difference: Actual	-2	
Difference: Hodges-Lehmann	-2	

Supplementary Table 7.

**Fig. 1l. Number of gold particles per synapse**

	His-tag::GluA2	His-tag::NR1
N (samples)	2	2
Number of values	47	40
Minimum	0	0
25% Percentile	5	3
Median	16	10.5
75% Percentile	27	21
Maximum	58	55
Range	58	55
95% CI of median		
Actual confidence level	96.00%	96.15%
Lower confidence limit	10	5
Upper confidence limit	20	14
Mean	17.87	13.78
Std. Deviation	15.45	13.48
Std. Error of Mean	2.254	2.132
Lower 95% CI of mean	13.34	9.463
Upper 95% CI of mean	22.41	18.09
Coefficient of variation	86.44%	97.89%

Skewness	0.9399	1.41
Kurtosis	0.1706	1.591
**Mann Whitney test**		
P value	0.1997	
Exact or approximate P value?	Exact	
P value summary	ns	
Significantly different (P < 0.05)?	No	
One- or two-tailed P value?	Two-tailed	
Sum of ranks in column A,B	2219 , 1609	
Mann-Whitney U	789	
Difference between medians		
Median of column A	16.00, n=47	
Median of column B	10.50, n=40	
Difference: Actual	-5.5	
Difference: Hodges-Lehmann	-3	

Supplementary Table 8.

**Fig. 2c. Cumulative relative frequency distribution of lateral distances from docked vesicles to the center of the active zone from 2-D profiles.**					
	no stim	5 ms	11 ms		

N (samples)	10	10	10		
Number of values	1034	856	933		
Minimum	0	0	0		
25% Percentile	0.25	0.2442	0.2545		
Median	0.5261	0.5033	0.5122		
75% Percentile	0.7996	0.7692	0.7701		
Maximum	0.9998	0.9978	0.9985		
Range	0.9998	0.9978	0.9985		
95% CI of median					
Actual confidence level	95.00%	95.63%	95.06%		
Lower confidence limit	0.4885	0.4629	0.4692		
Upper confidence limit	0.548	0.5285	0.5378		
Mean	0.5144	0.5043	0.5105		
Std. Deviation	0.3017	0.2966	0.2915		
Std. Error of Mean	0.009382	0.01014	0.0095 44		
Lower 95% CI of mean	0.496	0.4844	0.4917		
Upper 95% CI of mean	0.5328	0.5242	0.5292		
Coefficient of variation	58.65%	58.81%	57.11%		

Skewness	-0.05409	0.02533	0.0057 36		
Kurtosis	-1.273	-1.225	-1.221		
**one sample Wilcoxon test (against a theoretical median)**					
Theoretical median	0.5	0.5	0.5		
Actual median	0.526	0.503	0.512		
Number of values	1034	856	933		
Wilcoxon Signed Rank Test					
Sum of signed ranks (W)	30077	6406	18133		
Sum of positive ranks	282586	186601	226922		
Sum of negative ranks	-252509	-180195	- 208789		
P value (two tailed)	0.1174	0.6581	0.2708		
Exact or estimate?	Approximat e	Approximat e	Approximate		
P value summary	ns	ns	ns		
Significant (alpha=0.05)?	No	No	No		
How big is the discrepancy?					
Discrepancy	0.0261	0.00331	0.0122		
95% confidence interval	-0.0115 to 0.0480	-0.0371 to 0.0285	-0.0308 to 0.0378		

Actual confidence level	95	95.6	95.1		
**ANOVA summary**					
F	0.27				
P value	0.7634				
P value summary	ns				
Significant diff. among means (P < 0.05)?	No				
R squared	0.000191				
Brown-Forsythe test					
F (DFn, DFd)	1.03 (2, 2820)				
P value	0.3587				
P value summary	ns				
Are SDs significantly different (P < 0.05)?	No				
Bartlett's test					
Bartlett's statistic (corrected)	1.15				
P value	0.5623				
P value summary	ns				
Are SDs significantly different (P < 0.05)?	No				

ANOVA table	SS	DF	MS	F (DFn, DFd)	P value
Treatment (between columns)	0.0476	2	0.0238	F (2, 2820) = 0.270	P=0.7634
Residual (within columns)	248	2820	0.0881		
Total	249	2822			
Data summary					
Number of treatments (columns)	3				
Number of values (total)	2823				
**Tukey's multiple comparisons test**	Mean Diff.	95.00% CI of diff.	Significant?	Summary	Adjusted P Value
no stim vs. 5 ms	0.0101	-0.0221 to 0.0422	No	ns	0.7439
no stim vs. 11 ms	0.00392	-0.0275 to 0.0353	No	ns	0.954
5 ms vs. 11 ms	-0.00613	-0.0391 to 0.0268	No	ns	0.9003

Supplementary Table 9.

**Fig. 2d. Cumulative relative frequency distribution of lateral distances from exocytic pits to the center of the active zone from 2-D profiles.**

	5 ms	11 ms
N (samples)	6	6
Number of values	286	124
Minimum	0.004588	0
25% Percentile	0.195	0.05911
Median	0.404	0.1275
75% Percentile	0.5788	0.2292
Maximum	0.9956	0.9031
Range	0.991	0.9031
95% CI of median		
Actual confidence level	96.17%	96.16%
Lower confidence limit	0.3669	0.09333
Upper confidence limit	0.4398	0.1475
Mean	0.3984	0.1904
Std. Deviation	0.2392	0.2018
Std. Error of Mean	0.01415	0.01812
Lower 95% CI of mean	0.3706	0.1545
Upper 95% CI of mean	0.4263	0.2262
Coefficient of variation	60.04%	106.0%

Skewness	0.199	1.771
Kurtosis	-0.7496	2.682
**one sample Wilcoxon test (against a theoretical median)**		
Theoretical median	0.5	0.5
Actual median	0.404	0.128
Number of values	286	124
Wilcoxon Signed Rank Test		
Sum of signed ranks (W)	-18161	-7176
Sum of positive ranks	11440	287
Sum of negative ranks	-29601	-7463
P value (two tailed)	<0.0001	<0.0001
Exact or estimate?	Approximate	Exact
P value summary	****	****
Significant (alpha=0.05)?	Yes	Yes
How big is the discrepancy?		
Discrepancy	-0.096	-0.372
95% confidence interval	-0.133 to -0.0602	-0.407 to -0.353
Actual confidence level	96.2	96.2

**Kolmogorov-Smirnov test**		
P value	<0.0001	
Exact or approximate P value?	Approximate	
P value summary	****	
Significantly different (P < 0.05)?	Yes	
Kolmogorov-Smirnov D	0.4931	

Supplementary Table 10.

**Fig. 2e. Receptor locations relative to docked vesicles**									
**AMPA receptors**	no stim	5 ms	11 ms		**NMDA receptors**	no stim	5 ms	11 ms	
N (samples)	3	3	3		N (samples)	3	3	3	
Number of values	995	183	551		Number of values	317	203	174	
Minimum	0	0.74	0.94		Minimum	0.74	0	2.102	
25% Percentile	34.42	49.33	40.65		25% Percentile	34.97	23.13	37.8	
Median	94.91	101.6	92.41		Median	73.42	62.62	87.49	
75% Percentile	215.9	159.8	171.6		75% Percentile	130.7	142.3	188.7	

Maximum	799.7	609.6	595.3		Maximum	712.2	470.8	663.3	
Range	799.7	608.8	594.3		Range	711.4	470.8	661.2	
95% CI of median					95% CI of median				
Actual confidence level	95.07%	96.18%	95.01%		Actual confidence level	95.70%	95.09%	95.96%	
Lower confidence limit	86.13	77.75	81.92		Lower confidence limit	65.09	45.73	73.54	
Upper confidence limit	103.9	117.4	104.4		Upper confidence limit	86.97	81.69	111.1	
Mean	147.4	124.8	123.8		Mean	110.4	103.9	133.5	
Std. Deviation	148.4	111.3	106.8		Std. Deviation	120	109.5	134.2	
Std. Error of Mean	4.704	8.228	4.548		Std. Error of Mean	6.74	7.687	10.18	
Lower 95% CI of mean	138.1	108.6	114.9		Lower 95% CI of mean	97.19	88.74	113.4	

Upper 95% CI of mean	156.6	141.1	132.7		Upper 95% CI of mean	123.7	119	153.5	
Coefficient of variation	100.7%	89.17%	86.23%		Coefficient of variation	108.7%	105.4%	100.6%	
Skewness	1.485	1.65	1.16		Skewness	2.467	1.423	1.634	
Kurtosis	2.134	3.357	0.8445		Kurtosis	7.265	1.383	2.579	
**Kruskal-Wallis test**					**Kruskal-Wallis test**				
P value	0.6254				P value	0.034			
Exact or approximate P value?	Approximate				Exact or approximate P value?	Approximate			
P value summary	ns				P value summary	*			
Do the medians vary signif. (P < 0.05)?	No				Do the medians vary signif. (P < 0.05)?	Yes			

Number of groups	3				Number of groups	3			
Kruskal-Wallis statistic	0.9388				Kruskal-Wallis statistic	6.764			
**Dunn's multiple comparisons test**	Mean rank diff.	Significant?	Summary	Adjusted P Value	**Dunn's multiple comparisons test**	Mean rank diff.	Significant?	Summary	Adjusted P Value
no stim vs. 5ms	19.17	No	ns	>0.9999	no stim vs. 5ms	23.86	No	ns	0.5564
no stim vs. 11ms	24.7	No	ns	>0.9999	no stim vs. 11ms	-29.99	No	ns	0.3387
5ms vs. 11ms	5.532	No	ns	>0.9999	5ms vs. 11ms	-53.85	Yes	*	0.028

Supplementary Table 11.

**Fig. 2f. Receptor locations relative to pits**						
**AMPA receptors**	5 ms	11 ms		**NMDA receptors**	5 ms	11 ms
N (samples)	3	3		N (samples)	3	3
Number of values	118	37		Number of values	96	75

Minimum	1.88	40.41		Minimum	8.521	2.82
25% Percentile	24.31	92.06		25% Percentile	62.11	26.65
Median	67.14	120.1		Median	138.6	56.25
75% Percentile	148.8	160.1		75% Percentile	238.9	134.6
Maximum	436.2	258.7		Maximum	558.2	258.8
Range	434.3	218.3		Range	549.7	256
95% CI of median				95% CI of median		
Actual confidence level	96.62%	95.30 %		Actual confidence level	96.85%	96.30 %
Lower confidence limit	48.61	104		Lower confidence limit	94.39	36.25
Upper confidence limit	95.87	143.6		Upper confidence limit	171.5	89.57
Mean	98.85	124.9		Mean	155.4	83.75
Std. Deviation	97.03	50.78		Std. Deviation	112.2	71.39
Std. Error of Mean	8.932	8.348		Std. Error of Mean	11.45	8.243
Lower 95% CI of mean	81.16	108		Lower 95% CI of mean	132.7	67.32
Upper 95% CI of mean	116.5	141.8		Upper 95% CI of mean	178.2	100.2
Coefficient of variation	98.16%	40.66 %		Coefficient of variation	72.19%	85.25 %
Skewness	1.417	0.706 5		Skewness	0.8974	0.835

Kurtosis	1.773	0.5238		Kurtosis	0.6163	-0.4438
**Kolmogorov-Smirnov test**				**Kolmogorov-Smirnov test**		
P value	0.0002			P value	0.0006	
Exact or approximate P value?	Approximate			Exact or approximate P value?	Approximate	
P value summary	***			P value summary	***	
Significantly different (P < 0.05)?	Yes			Significantly different (P < 0.05)?	Yes	
Kolmogorov-Smirnov D	0.4052			Kolmogorov-Smirnov D	0.312	
**Mann Whitney test**				**Mann Whitney test**		
P value	0.0014			P value	<0.0001	
Exact or approximate P value?	Exact			Exact or approximate P value?	Exact	
P value summary	**			P value summary	****	
Significantly different (P < 0.05)?	Yes			Significantly different (P < 0.05)?	Yes	
One- or two-tailed P value?	Two-tailed			One- or two-tailed P value?	Two-tailed	
Sum of ranks in column A,B	8453 , 3637			Sum of ranks in column A,B	9727 , 4979	
Mann-Whitney U	1432			Mann-Whitney U	2129	

Supplementary Table 12.

**Fig. 3d. NMDA response to synchronous and asynchronous release events in the presence of Mg2+, while varing the timing of the asynchronous release**				
timing of asynchronous release	5 ms	8 ms	10 ms	20 ms
N (number of simulations)	48	48	48	48
area under curve (arbitary unit)	1527	1488	1357	1351

Supplementary Table 13.

**Fig. 3f. NMDA response to synchronous and asynchronous release events in the presence of Mg2+, while varing the locations of release (asynchronous release at 5 ms)**				
locations of synchronous release	AMPA R	NMDA R	NMDAR 15 mV	NMDAR 30 mV
locations of asynchronous release	NMDA R	AMPA R	NMDAR	NMDAR
N (number of simulations)	48	48	48	48
area under curve (arbitary unit)	1527	1527	1241	1725

Supplementary Table 14.

**Supplementary Fig. 1c. number of gold particles per synaptic profile**		

**wild type**	His-tag::GluA2	HaloTag::GluA2
N (samples)	4	3
Number of values	4	3
Number of images	385	214
Minimum	1.83	0.00952
25% Percentile	2	0.00952
Median	2.58	0.027
75% Percentile	3.8	0.153
Maximum	4.18	0.153
Range	2.35	0.143
95% CI of median		
Actual confidence level	87.5%	75.0%
Lower confidence limit	1.83	0.00952
Upper confidence limit	4.18	0.153
Mean	2.79	0.0632
Std. Deviation	0.992	0.0783
Std. Error of Mean	0.496	0.0452
Lower 95% CI of mean	1.21	-0.131
Upper 95% CI of mean	4.37	0.258
Coefficient of variation	35.6%	124%

Skewness	1.21	1.64
Kurtosis	2.27	
**Unpaired t test with Welch's correction**		
P value	0.0115	
P value summary	*	
Significantly different (P < 0.05)?	Yes	
One- or two-tailed P value?	Two-tailed	
Welch-corrected t, df	t=5.473, df=3.050	
How big is the difference?		
Mean of column A	2.79	
Mean of column B	0.06317	
Difference between means (B - A) ± SEM	-2.727 ± 0.4983	
95% confidence interval	-4.298 to -1.156	
R squared (eta squared)	0.9076	
F test to compare variances		
F, DFn, Dfd	160.7, 3, 2	
P value	0.0124	
P value summary	*	
Significantly different (P < 0.05)?	Yes	

Supplementary Table 15.

Supplementary Fig. 1d. Percentage of synaptic profile with gold particles		
**wild type**	His-tag::GluA2	HaloTag::GluA2
N (samples)	4	3
Number of values	4	3
Number of images	385	214
Minimum	64.4	0
25% Percentile	65.7	0
Median	69.5	2.7
75% Percentile	71.8	8.3
Maximum	72.5	8.3
Range	8.1	8.3
95% CI of median		
Actual confidence level	87.5%	75.0%
Lower confidence limit	64.4	0
Upper confidence limit	72.5	8.3
Mean	69	3.67
Std. Deviation	3.36	4.23
Std. Error of Mean	1.68	2.44
Lower 95% CI of mean	63.6	-6.85

Upper 95% CI of mean	74.3	14.2
Coefficient of variation	4.88%	115%
Skewness	-0.905	0.974
Kurtosis	1.95	
**Unpaired t test with Welch's correction**		
P value	<0.0001	
P value summary	****	
Significantly different (P < 0.05)?	Yes	
One- or two-tailed P value?	Two-tailed	
Welch-corrected t, df	t=22.01, df=3.777	
How big is the difference?		
Mean of column A	68.98	
Mean of column B	3.667	
Difference between means (B - A) ± SEM	-65.31 ± 2.967	
95% confidence interval	-73.74 to -56.88	
R squared (eta squared)	0.9923	
F test to compare variances		
F, DFn, Dfd	1.585, 2, 3	
P value	0.6781	
P value summary	ns	

Significantly different (P < 0.05)?	No	

Supplementary Table 16.

Supplementary Fig. 1e. number of gold particles per synaptic profile		
**knockout**	His-tag::GluA2	HaloTag::GluA2
N (samples)	3	2
Number of values	3	2
Number of images	353	165
Minimum	2.11	0.00962
25% Percentile	2.11	0.00962
Median	2.19	0.054
75% Percentile	2.66	0.0984
Maximum	2.66	0.0984
Range	0.55	0.0888
95% CI of median		
Actual confidence level	75.0%	50.0%
Lower confidence limit	2.11	0.00962
Upper confidence limit	2.66	0.0984
Mean	2.32	0.054

Std. Deviation	0.297	0.0628
Std. Error of Mean	0.172	0.0444
Lower 95% CI of mean	1.58	-0.51
Upper 95% CI of mean	3.06	0.618
Coefficient of variation	12.8%	116%
Skewness	1.59	
Kurtosis		
**Unpaired t test with Welch's correction**		
P value	0.0038	
P value summary	**	
Significantly different (P < 0.05)?	Yes	
One- or two-tailed P value?	Two-tailed	
Welch-corrected t, df	t=12.79, df=2.257	
How big is the difference?		
Mean of column A	2.32	
Mean of column B	0.05401	
Difference between means (B - A) ± SEM	-2.266 ± 0.1772	
95% confidence interval	-2.951 to -1.581	
R squared (eta squared)	0.9864	

Supplementary Table 17.

**Supplementary Fig. 1f. Percentage of synaptic profile with gold particles**		
**knockout**	His-tag::GluA2	HaloTag::GluA2
N (samples)	3	2
Number of values	3	2
Number of images	353	165
Minimum	56.4	0
25% Percentile	56.4	0
Median	62.9	4.9
75% Percentile	75	9.8
Maximum	75	9.8
Range	18.6	9.8
95% CI of median		
Actual confidence level	75.0%	50.0%
Lower confidence limit	56.4	0
Upper confidence limit	75	9.8
Mean	64.8	4.9
Std. Deviation	9.44	6.93
Std. Error of Mean	5.45	4.9

Lower 95% CI of mean	41.3	-57.4
Upper 95% CI of mean	88.2	67.2
Coefficient of variation	14.6%	141%
Skewness	0.855	
Kurtosis		
**Unpaired t test with Welch's correction**		
P value	0.0047	
P value summary	**	
Significantly different (P < 0.05)?	Yes	
One- or two-tailed P value?	Two-tailed	
Welch-corrected t, df	t=8.169, df=2.835	
How big is the difference?		
Mean of column A	64.77	
Mean of column B	4.9	
Difference between means (B - A) ± SEM	-59.87 ± 7.329	
95% confidence interval	-83.98 to -35.76	
R squared (eta squared)	0.9592	

Supplementary Table 18.

Supplementary Fig. 2c. Cumulative relative frequency distribution of lateral distances from receptors to the center of the active zone from 2-D profiles.						
	ampa first 25	nmda first 25	ampa first 100	nmda first 100	ampar total	nmdar total
Number of values	25	25	100	100	808	551
Minimum	0.093	0.03	0.0423	0.0174	0	0
25% Percentile	0.296	0.239	0.267	0.204	0.277	0.149
Median	0.639	0.649	0.526	0.416	0.569	0.33
75% Percentile	0.789	0.878	0.848	0.664	0.819	0.508
Maximum	0.959	0.982	0.989	0.982	1	0.991
Range	0.866	0.952	0.947	0.964	1	0.991
95% CI of median						
Actual confidence level	95.7%	95.7%	96.5%	96.5%	95.5%	95.0%
Lower confidence limit	0.334	0.346	0.397	0.356	0.541	0.296
Upper confidence limit	0.738	0.856	0.683	0.503	0.608	0.355
Mean	0.559	0.555	0.536	0.443	0.547	0.348
Std. Deviation	0.286	0.332	0.311	0.276	0.297	0.24
Std. Error of Mean	0.0573	0.0665	0.0311	0.0276	0.0105	0.0102

Lower 95% CI of mean	0.441	0.418	0.474	0.389	0.526	0.328
Upper 95% CI of mean	0.677	0.692	0.598	0.498	0.567	0.368
Coefficient of variation	51.2%	59.8%	58.1%	62.3%	54.4%	69.1%
Skewness	-0.188	-0.241	-0.0385	0.293	-0.196	0.574
Kurtosis	-1.32	-1.38	-1.44	-0.955	-1.27	-0.355
**one sample Wilcoxon test (against a theoretical median)**						
Theoretical median	0.5	0.5	0.5	0.5	0.5	0.5
Actual median	0.639	0.649	0.526	0.416	0.569	0.33
Number of values	25	25	100	100	808	551
Wilcoxon Signed Rank Test						
Sum of signed ranks (W)	79	63	754	-1182	58476	-94302
Sum of positive ranks	202	194	2902	1934	192656	28887
Sum of negative ranks	-123	-131	-2148	-3116	-134180	-123189

P value (two tailed)	0.2996	0.4068	0.1961	0.0419	<0.0001	<0.0001
Exact or estimate?	Exact	Exact	Exact	Exact	Approximate	Approximate
P value summary	ns	ns	ns	*	****	****
Significant (alpha=0.05)?	No	No	No	Yes	Yes	Yes
How big is the discrepancy?						
Discrepancy	0.139	0.149	0.0264	-0.0837	0.0689	-0.17
95% confidence interval	-0.166 to 0.238	-0.154 to 0.356	-0.103 to 0.183	-0.144 to 0.00254	0.0407 to 0.108	-0.204 to -0.145
Actual confidence level	95.7	95.7	96.5	96.5	95.5	95

Supplementary Table 19.

**Supplementary Fig. 3e. Percentage of synaptic profile with gold particles**		
	His-tag::GluA2	SnapTag::GluA2
Number of cultures	3	2
Number of values	9	6
Number of images	934	834

Minimum	0.434	0.0286
25% Percentile	0.468	0.0429
Median	0.63	0.089
75% Percentile	0.697	0.216
Maximum	0.722	0.315
Range	0.288	0.286
95% CI of median		
Actual confidence level	96.1%	96.9%
Lower confidence limit	0.455	0.0286
Upper confidence limit	0.721	0.315
Mean	0.591	0.125
Std. Deviation	0.112	0.108
Std. Error of Mean	0.0373	0.044
Lower 95% CI of mean	0.505	0.0122
Upper 95% CI of mean	0.677	0.238
Coefficient of variation	18.9%	86.0%
Skewness	-0.31	1.3
Kurtosis	-1.6	1.18

**Unpaired t test with Welch's correction**		
P value	<0.0001	
P value summary	****	
Significantly different (P < 0.05)?	Yes	
One- or two-tailed P value?	Two-tailed	
Welch-corrected t, df	t=8.08, df=11.2	
How big is the difference?		
Mean of column A	0.591	
Mean of column B	0.125	
Difference between means (B - A) ± SEM	-0.466 ± 0.0577	
95% confidence interval	-0.593 to -0.339	
R squared (eta squared)	0.854	
F test to compare variances		
F, DFn, Dfd	1.08, 8, 5	
P value	0.9803	
P value summary	ns	
Significantly different (P < 0.05)?	No	

Supplementary Table 20.

**Supplementary Fig. 3f. Percentage of synaptic profile with gold particles**		

	His-tag::NR1	SnapTag::NR1
Number of cultures	3	2
Number of values	9	3
Number of images	836	584
Minimum	0.524	0
25% Percentile	0.529	0.0144
Median	0.574	0.0955
75% Percentile	0.656	0.25
Maximum	0.828	0.297
Range	0.303	0.297
95% CI of median		
Actual confidence level	96.1%	96.9%
Lower confidence limit	0.529	0
Upper confidence limit	0.718	0.297
Mean	0.603	0.124
Std. Deviation	0.103	0.126
Std. Error of Mean	0.0343	0.0513
Lower 95% CI of mean	0.524	-0.00825
Upper 95% CI of mean	0.682	0.255
Coefficient of variation	17.1%	102%

Skewness	1.67	0.42
Kurtosis	2.12	-2.06
**Unpaired t test with Welch's correction**		
P value	<0.0001	
P value summary	****	
Significantly different (P < 0.05)?	Yes	
One- or two-tailed P value?	Two-tailed	
Welch-corrected t, df	t=7.77, df=9.32	
How big is the difference?		
Mean of column A	0.603	
Mean of column B	0.124	
Difference between means (B - A) ± SEM	-0.480 ± 0.0617	
95% confidence interval	-0.619 to -0.341	
R squared (eta squared)	0.866	
F test to compare variances		
F, DFn, Dfd	1.49, 5, 8	
P value	0.5879	
P value summary	ns	
Significantly different (P < 0.05)?	No	

Supplementary Table 21.

**Supplementary Fig. 3g. Number of docked vesicles per synaptic profile**					
Number of cultures	3	3	3		
Number of values	10	10	10		
Number of images	740	835	791		
Minimum	1.72	0.726	0.685		
25% Percentile	1.77	0.816	0.745		
Median	1.87	0.919	0.902		
75% Percentile	1.92	0.995	0.958		
Maximum	2.06	1.2	1.16		
Range	0.34	0.474	0.47		
95% CI of median					
Actual confidence level	97.9%	97.9%	97.9%		
Lower confidence limit	1.75	0.74	0.714		
Upper confidence limit	2.01	1.01	1.03		
Mean	1.87	0.919	0.883		
Std. Deviation	0.106	0.138	0.145		
Std. Error of Mean	0.0336	0.0437	0.0458		

Lower 95% CI of mean	1.79	0.82	0.78		
Upper 95% CI of mean	1.94	1.02	0.987		
Coefficient of variation	5.69%	15.0%	16.4%		
Skewness	0.463	0.538	0.392		
Kurtosis	-0.101	0.934	-0.112		
**Two-way ANOVA**	Ordinary				
Alpha	0.05				
Source of Variation	% of total variation	P value	P value summary	Significant?	
Interaction	1.172	0.0004	***	Yes	
Row Factor	0.5597	0.0107	*	Yes	
Column Factor	20.02	<0.0001	****	Yes	
ANOVA table	SS (Type III)	DF	MS	F (DFn, DFd)	P value
Interaction	31.31	18	1.739	F (18, 2921) = 2.505	P=0.0004
Row Factor	14.94	9	1.66	F (9, 2921) = 2.392	P=0.0107

Column Factor	534.5	2	267.2	F (2, 2921) = 384.9	P<0.0001
Residual	2028	2921	0.6942		
**Tukey's multiple comparisons test**	Predicted (LS) mean diff.	95.00% CI of diff.	Significant?	Summary	Adjusted P Value
no stim vs. 5 ms	0.9488	0.8569 to 1.041	Yes	****	<0.0001
no stim vs. 11 ms	0.9841	0.8910 to 1.077	Yes	****	<0.0001
5 ms vs. 11 ms	0.0353	-0.05155 to 0.1221	No	ns	0.6066

Supplementary Table 22.

**Supplementary Fig. 3h. Number of pits per synaptic profile**					
Number of cultures	3	3	3		
Number of values	10	10	10		
Number of images	740	835	791		
Number of values	10	10	10		

Minimum	0	0.0885	0.02655		
25% Percentile	0	0.2248	0.07657		
Median	0.01984	0.2961	0.1195		
75% Percentile	0.02844	0.3247	0.1606		
Maximum	0.04808	0.4623	0.2667		
Range	0.04808	0.3738	0.2402		
95% CI of median					
Actual confidence level	97.85%	97.85%	97.85%		
Lower confidence limit	0	0.2	0.06863		
Upper confidence limit	0.02885	0.3558	0.2135		
Mean	0.01782	0.2794	0.1249		
Std. Deviation	0.01563	0.09906	0.07061		
Std. Error of Mean	0.004944	0.03133	0.02233		
Lower 95% CI of mean	0.006632	0.2085	0.07439		
Upper 95% CI of mean	0.029	0.3502	0.1754		
Coefficient of variation	87.75%	35.46%	56.54%		
Skewness	0.4709	-0.1392	0.8931		
Kurtosis	-0.05549	1.395	0.7211		

**Two-way ANOVA**	Ordinary				
Alpha	0.05				
Source of Variation	% of total variation	P value	P value summary	Significant?	
Interaction	1.781	<0.0001	****	Yes	
Row Factor	1.316	<0.0001	****	Yes	
Column Factor	7.814	<0.0001	****	Yes	
ANOVA table	SS (Type III)	DF	MS	F (DFn, DFd)	P value
Interaction	7.298	18	0.4055	F (18, 2921) = 3.272	P<0.0001
Row Factor	5.392	9	0.5991	F (9, 2921) = 4.835	P<0.0001
Column Factor	32.02	2	16.01	F (2, 2921) = 129.2	P<0.0001
Residual	362	2921	0.1239		
**Tukey's multiple comparisons test**	Predicted (LS) mean diff.	95.00% CI of diff.	Significant ?	Summary	Adjusted P Value
no stim vs. 5 ms	-0.2616	-0.3004 to -0.2227	Yes	****	<0.0001

no stim vs. 11 ms	-0.1071	-0.1464 to -0.06774	Yes	****	<0.0001
5 ms vs. 11 ms	0.1545	0.1178 to 0.1912	Yes	****	<0.0001

Supplementary Table 23.

**Supplementary Fig. 4c. AMPAR response to synchronous and asynchronous release events in the absence of Mg2+, while varing the timing of the asynchronous release**									
locations of synchronous release	random	NMDAR	AMPAR	AMPAR+NMDAR	AMPAR	AMPAR	AMPAR	AMPAR	AMPAR
locations of asynchronous release	-	-	-	-	NMDAR	NMDAR	NMDAR	NMDAR	NMDAR
timing of asynchronous release (ms)	-	-	-	-	5	10	15	20	50

N (number of simulations)	48	48	48	48	48	48	48	48	48
area under curve (arbitary unit)	3431	3267	4581.271	6857.813	6667.521	6807.447	7070.021	7325	8006.625

Supplementary Table 24.

**Supplementary Fig. 4f. NMDAR response to synchronous and asynchronous release events in the absence of Mg2+, while varing the timing of the asynchronous release**									
locations of synchronous release	random	NMDAR	AMPAR	AMPAR+NMDAR	AMPAR	AMPAR	AMPAR	AMPAR	AMPAR
locations of asynchronous release	-	-	-	-	NMDAR	NMDAR	NMDAR	NMDAR	NMDAR
timing of asynchronous release (ms)	-	-	-	-	5	10	15	20	50
N (number of simulations)	48	48	48	48	48	48	48	48	48

area under curve (arbitary unit)	6970	24470	26367.02	48406.75	53986.9	53172.98	55250.13	54429.69	58565.92

Supplementary Table 25.

**Supplementary Fig. 4j. NMDAR response to synchronous and asynchronous release events in the absence of Mg2+, while varing the locations of release, the timing of the asynchronous release and the membrane potentials**									
locations of synchronous release	AMPAR+ NMDAR	AMPAR	NMDAR	NMDAR	NMDAR	NMDAR	NMDAR	NMDAR	NMDAR
depolarization during synchronous (mV)	45	30	0	15	15	30	30	30	30
locations of asynchronous release	-	NMDAR	AMPAR	AMPAR	NMDAR	NMDAR	NMDAR	NMDAR	NMDAR
depolarization during asynchronous (mV)	-	15	30	30	15	15	15	15	15
timing of asynchronous release (ms)	-	5	5	5	5	5	8	10	20
N (number of simulations)	48	48	48	48	48	48	48	48	48
area under curve (arbitary unit)	1725	1527	1293.468	1527	1241	1725	1586	1631	1544

Supplementary Table 26.

**Supplementary Fig. 4g. AMPAR and NMDAR response to single or double release events in the absence of Mg2+, while varing the locations of release**				
locations of synchronous release	AMPAR	NMDAR	random	AMPAR
locations of asynchronous release	-	-	-	NMDAR
NMDAR response	26367	24470	12800	53986
AMPAR respponse	4581	3267	3431	6667
NMDA/AMPA ratio	5.76	7.49	3.73	8.09
N (number of simulations)	48	48	48	48
