# Supplementary material for: Asynchronous release sites align with NMDA receptors in mouse hippocampal synapses
Source: Nat Commun. 2021 Jan 29;12:677. doi: 10.1038/s41467-021-21004-x (PMC7846561; doi:10.1038/s41467-021-21004-x)
Supplement: Supplementary file 3 — Description of Additional Supplementary Files [file 41467_2021_21004_MOESM3_ESM.pdf]

Description of Additional Supplementary Files

Title: Supplementary Movie 1

Description: An example movie showing the serial electron micrographs of a synapse, expressing His-tag::GluA2, after treatment with Ni-NTA-gold.

Title: Supplementary Movie 2

Description: An example movie showing the serial electron micrographs of a synapse, expressing His-tag::GluA2, after treatment with Ni-NTA-gold.

Title: Supplementary Movie 3

Description: An example movie showing the serial electron micrographs of a synapse, expressing His-tag::NR1, after treatment with Ni-NTA-gold.

Title: Supplementary Movie 4

Description: An example movie showing the serial electron micrographs of a synapse, expressing His-tag::NR1, after treatment with Ni-NTA-gold.
